# New efficient ligand for sub-mol % copper-catalyzed C–N cross-coupling reactions running under air

**DOI:** 10.3762/bjoc.8.221

**Published:** 2012-11-09

**Authors:** Per-Fredrik Larsson, Peter Astvik, Per-Ola Norrby

**Affiliations:** 1 University of Gothenburg, Department of Chemistry and Molecular Biology, Kemigården 4, SE-412 96, Gothenburg, Sweden

**Keywords:** copper, cross coupling, hyperactivity, kinetics, ligands

## Abstract

A new efficient ligand, *N*,*N*’’-dimethyldiethylene triamine (DMDETA), has been synthesized and evaluated for sub-mol % copper-catalyzed C–N cross-coupling reactions. The efficiency of the ligand was determined by kinetic methods. DMDETA proved to display efficiency similar to DMEDA and, in addition, the resulting catalyst was tolerant to air.

## Introduction

Copper-catalyzed heteroatom cross-coupling reactions (C–N, C–O and C–S) are important synthetic tools in modern organic synthesis [1–6]. Even though copper salts have been known to catalyze these transformations for a long period of time (i.e., the Ullmann [7] and Goldberg reactions [8]) the reaction type has had a reputation of being inefficient, requiring high temperature and high catalytic loading (near stoichiometric) [1]. However, the revival of copper catalysis by Buchwald and co-workers [9–24] and others [25–75] during the 1990s and 2000s proved that copper is a competitive catalyst alongside the more common choices such as palladium and nickel. With a few exceptions [23,45], the catalytic loading is still in the range of 5–20 mol %. We have shown recently that in many cases sub-mol % of copper is enough to catalyze several transformations [76–77]. By keeping the ligand (DMEDA) concentration high (>20 mol %) the cross-coupling reactions can be run with copper concentrations down to 0.001 mol % (10 ppm). The temperature can be lowered to 65 °C if the reaction is run in neat DMEDA. Using low catalytic loadings also eliminates the issue of arylation of the ligand, which is a known side-reaction when using higher catalytic loadings with secondary amine ligands [12–13]. Several classes of ligands have proved efficient in copper-catalyzed cross-coupling reactions, such as phenanthrolines, alpha-amino acids, 1,3-diketones, salicylamides, and imines, but DMEDA is one of the few that works well when sub-mol % copper is used [10,19,23,25,29,45,78–84].

## Results and Discussion

**Scheme 1:**
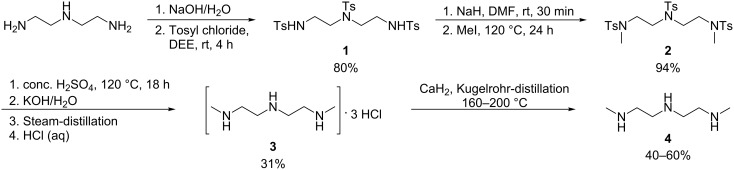
Synthetic procedure for *N*,*N*''-dimethyldiethylene triamine.

Although DMEDA has proved to be a very efficient ligand for C–N, C–O and C–S, some issues still remain, such as the sensitivity to air. As the Me–NH–ethylene–NH motif seemed to be necessary when lowering the copper concentration, dimethyldiethylene triamine (DMDETA) was a natural choice as ligand. The ligand was synthesized based on literature procedures starting from diethylene triamine (Scheme 1) [85–86]. Reductive methylation by using formaldehyde and NaBH_4_, as well as BOC-protection followed by reduction by LiAlH_4_, were also evaluated as potential synthetic routes, but both methods proved unsuccessful. In our hands, protecting all nitrogens followed by methylation of the two remaining free valencies on N was the most efficient method for synthesizing DMDETA.

The removal of the tosyl groups proved to be complicated due to issues with isolation of DMDETA during the workup procedure. Steam-distillation followed by acidification by HCl (aq) to produce the DMDETA trihydrochloride-salt **3** did solve the problem, but at the expense of lower yield. Distillation over CaH_2_ gave the pure DMDETA (**4**) in moderate yield.

**Scheme 2:**
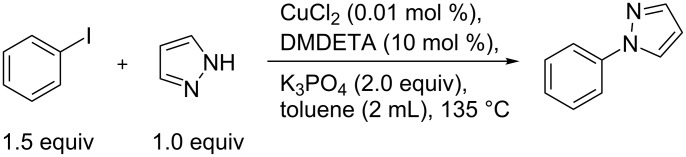
Standard reaction for the kinetic study.

To test the efficiency of DMDETA a kinetic study was performed by varying the concentration of the ligand and copper. As a standard reaction the copper-catalyzed cross coupling of pyrazole with iodobenzene was chosen (Scheme 2).

**Figure 1:**
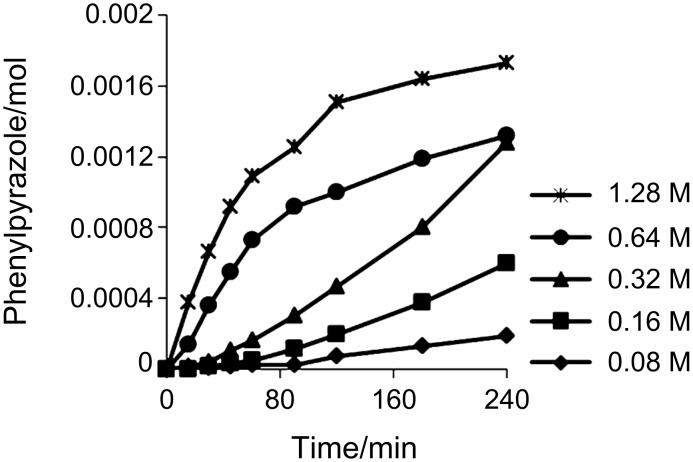
Kinetic profile for varying [DMDETA].

The kinetic profile when varying the concentration of DMDETA proved to be more or less the same as for our previously reported results concerning DMEDA (Figure 1) [77]

**Figure 2:**
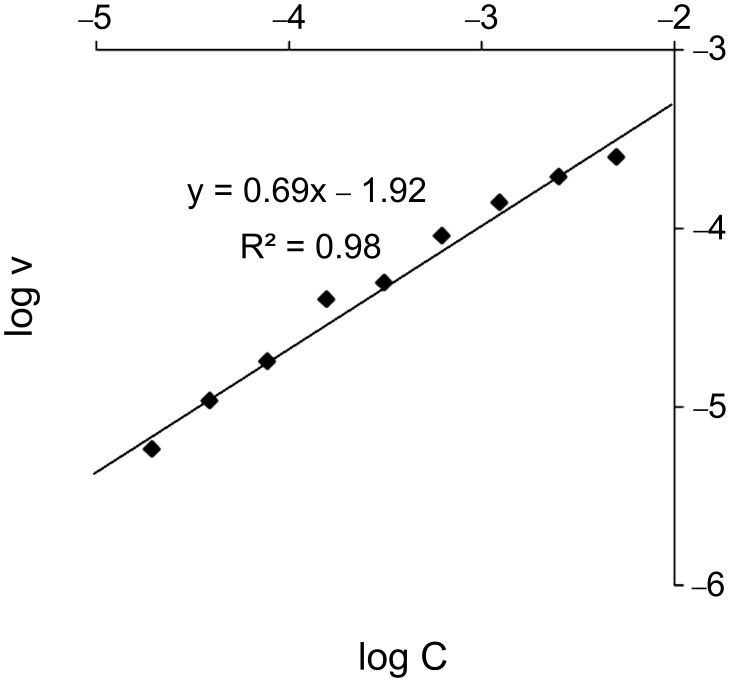
Reaction order for copper from 0.025–0.64 mol %.

The reaction order in copper was determined to be 0.7 (Figure 2). This is different from DMEDA, where the order was found to be 1 at Cu concentrations below ca. 1 mM, and 0 at higher concentrations [77]. A value lower than 1 indicates that an active monomeric catalyst is in equilibrium with inactive dimers or higher oligomers. The reaction order never drops to zero, as it did with DMEDA, showing that the formation of insoluble aggregates at high concentration [87] is avoided.

**Figure 3:**
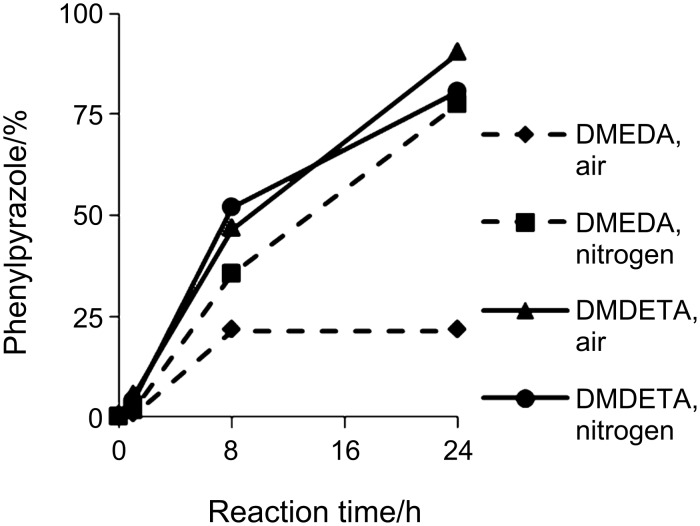
DMEDA versus DMDETA under air and nitrogen atmosphere.

Since DMDETA seems superior to DMEDA in solubilizing the catalyst and hence generating a more stable active catalyst, we decided to challenge the reaction with conditions where DMEDA fails, by testing the reaction without an inert atmosphere. Somewhat to our surprise, we found that the DMDETA-based catalyst was efficient even under air (Figure 3).

**Scheme 3:**
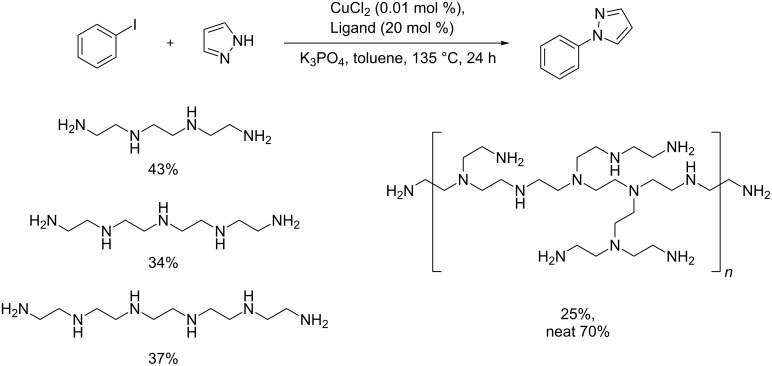
Long-chained aliphatic amines as ligands.

As can be seen from Figure 3, the standard reaction run with DMEDA is greatly hampered by the presence of air and stops after 8 h compared to that with DMDETA, which gives 90% yield after 24 h under the same conditions. To further investigate the ligand scope a range of long-chained aliphatic amines were evaluated in the standard reaction (Scheme 3).

None of the ligands in Scheme 3 showed satisfactory performance except neat polyethyleneimine (70% yield). A few selected solid-supported aliphatic amines (StratoSphere™ PL-EDA resin, StratoSphere™ PL-DETA, tris(2-aminoethyl)amine polymer-bound) were also tested as ligands but gave zero yield under standard reaction conditions. The fact that StratoSphere™ PL-DETA did not give any yield compared to the free amine (DETA, 41% yield after 24 h) [78] indicates that the amine needs to be in its free form and could be involved in the mass transfer of the heterogeneous base.

## Conclusion

In summary, a new and efficient ligand (DMDETA) for sub-mol % copper-catalyzed C–N cross coupling has been synthesized and evaluated in kinetic studies. DMDETA, together with DMEDA, is one of the few reported ligands that work at sub-mol % levels of copper catalyst [23,45,77–78,88]. DMDETA proved to be at least as efficient as DMEDA and in addition has the ability to render the catalyst tolerant to air. The reaction order of copper (0.7) lacked the nonlinear behavior previously reported for DMEDA [78], indicating that the ligand is more effective at solvating the active catalyst. The fact that DMDETA proved to be an effective ligand in sub-mol % copper catalysis further indicates the necessity for the Me–N–ethylene–N–Me motif in the ligand design of these systems. However, in our hands the synthetic procedures for mono-methylating ethylene amines were ineffective for the ethylenediamine motif; hence, there is an opportunity for further ligand synthesis development.

## Experimental

**General:** All the experiments were carried out under inert atmosphere (nitrogen). DMEDA, iodobenzene and dodecane were distilled over calcium hydride. Toluene was dried through distillation and stored under nitrogen. DMF was distilled over calcium hydride and stored under nitrogen. CuCl_2_ (Aldrich, purity of 99.999% metal basis), K_3_PO_4_ (98%), and pyrazole (98%) were stored under air- and moisture-free conditions. All other chemicals were used as received. A gas chromatograph with flame ionization detector and a 30 m × 0.25 mm EQUITY-5 fused silica capillary column was used, with hydrogen as carrier gas. General temperature program: 100 °C for 14 min, then up to 300 °C at 50 °C min^−1^ for 2 min. Dodecane was used as an internal standard. The NMR spectroscopic analysis was carried out on a Varian 400 MHz instrument by using CDCl_3_ or D_2_O as solvent, with shifts measured against TMS.

### Synthesis of *N*,*N*’’-dimethyldiethylene triamine (DMDETA)

**4-Methyl-*N*,*N*-bis(2-(4-methylphenylsulfonamido)ethyl)benzenesulfonamide (1):** Diethylene triamine (10 g, 97 mmol, 1 equiv) and NaOH (11.65 g, 291 mmol, 3 equiv) were added to a round-bottom flask, and H_2_O was added under stirring until the hydroxide was dissolved. Tosyl chloride (57.2 g, 300 mmol, 3.1 equiv) was dissolved in DEE (300 mL) and added over 1 h to the reaction mixture, followed by stirring at ambient temperature for 4 h. The resulting white solid was collected by filtration, washed repeatedly with hot water, and then with EtOH (3 × 50 mL), and dried in vacuo to yield **1** (44 g, 80%). ^1^H NMR (400 MHz, CDCl_3_) δ 2.43 (s, 9H), 3.16 (m, 8H), 7.31–7.77 (m, 12H) ppm; ^13^C NMR (400 MHz, CDCl_3_) δ 21.55 (3C), 42.65 (2C), 50.56 (2C), 127.16–127.31 (6C), 129.83–130.00 (6C), 134.67 (2C), 136.63 (3C), 143.65 (1C) ppm.

***N*,*N*-bis(2-(*N*,4-dimethylphenylsulfonamido)ethyl)-4-methylbenzenesulfonamide (2): 1** (44 g, 78 mmol, 1 equiv) was dissolved in dry DMF (500 mL) whereupon NaH (9.3 g, 234 mmol, 60% susp., 3 equiv) was added in portions under stirring, and stirring was then continued for 30 min at ambient temperature. Excess NaH was separated by allowing the solids to settle, whereupon the solution was removed with a syringe. The solution was heated to 120 °C. Iodomethane (15 mL, 234 mmol, 3 equiv) was added slowly under stirring at 120 °C and then heated under reflux for 24 h. Half of the solvent was evaporated, whereupon water was added (400 mL), resulting in a white/yellow precipitation. The solids were collected by filtration, washed repeatedly with hot water, and dried in vacuo to yield **2** (43.48 g, 94%). ^1^H NMR (400 MHz, CDCl_3_) δ 2.43 (s, 9H), 2.78 (s, 6H), 3.29 (m, 8H), 7.33 (m, 6H), 7.69 (m, 6H) ppm; ^13^C NMR (400 MHz, CDCl_3_) δ 21.53 (3C), 36.28 (2C), 48.22 (2C), 49.74 (2C), 127.40–127.51 (6C), 129.82–129.91 (6C), 133.94 (2C), 135.30 (3C), 143.61–143.87 (1C) ppm.

***N*,*N*’’-dimethyldiethylene triamine trihydrochloride (3): 2** (43.48 g, 73 mmol) was dissolved in conc. H_2_SO_4_ (90 mL) under heating, with continued stirring at 120 °C for 18 h. A saturated KOH/H_2_O solution was added slowly to the reaction mixture (cooled on ice) until it was strongly alkaline. The mixture was then steam-distilled, and the distillate was acidified with HCl and evaporated to dryness to yield the HCl salt of dimethyldiethylene triamine (12 g, crude). The salt was further purified by heating under reflux in EtOH (99.8%), and, upon cooling on ice, was filtrated and washed with ice cold EtOH to give the pure HCl-salt of *N*,*N*’’-dimethyldiethylene triamine (**3**) (5.28 g, 31%). ^1^H NMR (400 MHz, D_2_O) δ 2.69 (s, 6H), 3.38 (m, 8H) ppm; ^13^C NMR (400 MHz, D_2_O) δ 33.23 (2C), 43.32 (2C), 44.06 (2C) ppm.

***N*,*N*’’-dimethyldiethylene triamine (4):** The HCl-salt **3** was distilled over CaH_2_ by Kugelrohr distillation, at 160–200 °C under vacuum, to yield a clear oil of **4** (40–60%). ^1^H NMR (400 MHz, CDCl_3_) δ 1.22 (s, 3H), 2.39 (s, 6H), 2.68 (m, 8H) ppm; ^13^C NMR (400 MHz, CDCl_3_) δ 36.45 (2C), 49.27 (2C), 51.71 (2C) ppm.

**General procedure for the kinetic experiments:** The initial rate log plots were constructed from data points ranging from 0 to a maximum of 25% yield. The kinetic investigation was carried out by varying the concentration of one component and keeping the rest of the components constant under standard reaction conditions; pyrazole (136 mg, 2 mmol, 1 equiv), iodobenzene (334 μL, 1.5 equiv), CuCl_2_ (40 μL, 0.01 mol %) K_3_PO_4_ (849 mg, 2 equiv), DMDETA (26 mg, 0.2 mmol, 10 mol %) and dodecane (50 μL, 0.22 mmol). Into a microwave vial was added pyrazole (A mmol) and K_3_PO_4_ (B mmol). The vial was sealed and a CuCl_2_ solution (C μL, 5 mM in dry THF) was added. The THF was removed by three cycles of vacuum followed by nitrogen, whereupon toluene (2 mL), DMEDA (D mmol), iodobenzene (E mmol), and dodecane (50 mL, 0.22 mmol) were added. The closed vial was placed in a preheated aluminum block at 135 °C. Samples (50 μL) were collected at intervals, filtered through a small silica plug, and analyzed by GC. The yield from the GC was determined by using dodecane as the internal standard.

**Procedure for running under air or nitrogen:** The same procedure as the kinetic experiment was used; however, the reactions were not run in closed vials but under reflux in air or nitrogen. In this case 20 mol % of the ligands were used, DMEDA (43 μL, 0.4 mmol, 20 mol %) and DMDETA (52 mg, 0.4 mmol, 20 mol %). Samples were taken (50 μL) at certain time intervals by using the same workup procedure as mentioned above. The yield from the GC was determined by using dodecane as the internal standard.

**Procedure for ligand screening:** The same procedure as the kinetic experiment was used. 20 mol % of triethylene tetraamine (58 mg, 0.4 mmol), tetraethylene pentaamine (76 mg, 0.4 mmol), pentaethylene hexamine (93 mg, 0.4 mmol), polyethyleneimine (MW ≈ 800) (320 mg, 0.4 mmol), StratoSphere™ PL-EDA resin (80 mg, 0.4 mmol, 5–6 mmol g^−1^), StratoSphere™ PL-DETA (67 mg, 0.4 mmol, 6 mmol g^−1^), tris(2-aminoethyl)amine polymer-bound (114 mg, 0.4 mmol, 3.5–5 mmol g^−1^). Samples (50 μL) were taken after 24 h by using the same workup procedure as previously mentioned. The yield from the GC was determined by using dodecane as the internal standard.

## Supporting Information

File 1 ^1^H NMR and ^13^C NMR data of compounds **1**–**4**.
