# Supplementary material for: New efficient ligand for sub-mol % copper-catalyzed C–N cross-coupling reactions running under air
Source: Beilstein J Org Chem. 2012 Nov 9;8:1909–15. doi: 10.3762/bjoc.8.221 (PMC3511030; doi:10.3762/bjoc.8.221)

Supporting Information

for

New efficient ligand for sub-mol % copper-catalyzed C–N cross-coupling reactions running under air

Per-Fredrik Larsson, Peter Astvik, Per-Ola Norrby*

Address: University of Gothenburg, Department of Chemistry and Molecular Biology, Kemigården 4, SE-412 96, Gothenburg, Sweden

Email: Per-Ola Norrby - pon@chem.gu.se

* Corresponding author

^{1}H NMR and ^{13}C NMR data of compounds **1**–**4**.

^{1}H NMR (400 MHz, $CDCl_3$):

4-Methyl-*N*,*N*-bis(2-(4-methylphenylsulfonamido)ethyl)benzenesulfonamide (**1**)

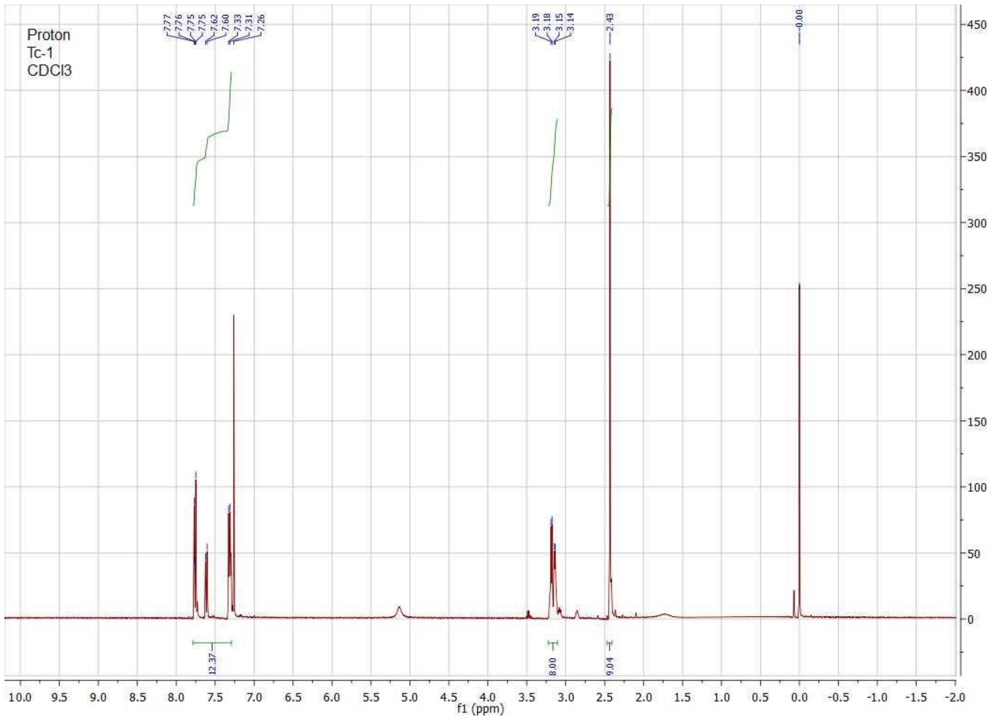

^{13}C NMR (400 MHz, $CDCl_3$):

4-Methyl-*N*,*N*-bis(2-(4-methylphenylsulfonamido)ethyl)benzenesulfonamide (**1**)

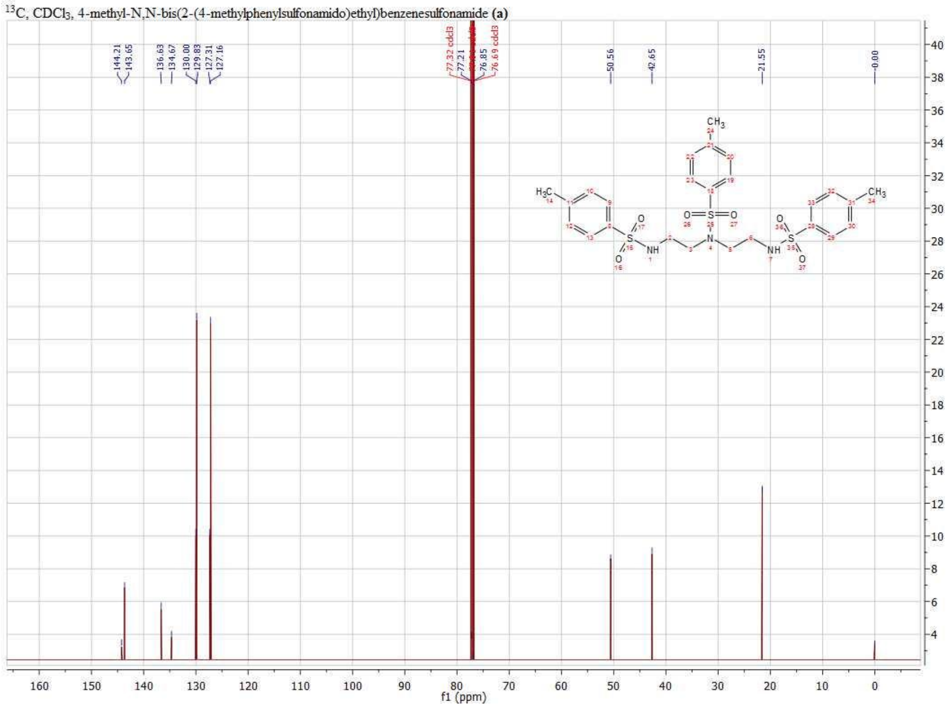

^{1}H NMR (400 MHz, $CDCl_3$):

N,*N*-Bis(2-(*N*,4-dimethylphenylsulfonamido)ethyl)-4-methylbenzenesulfon-amide (**2**)

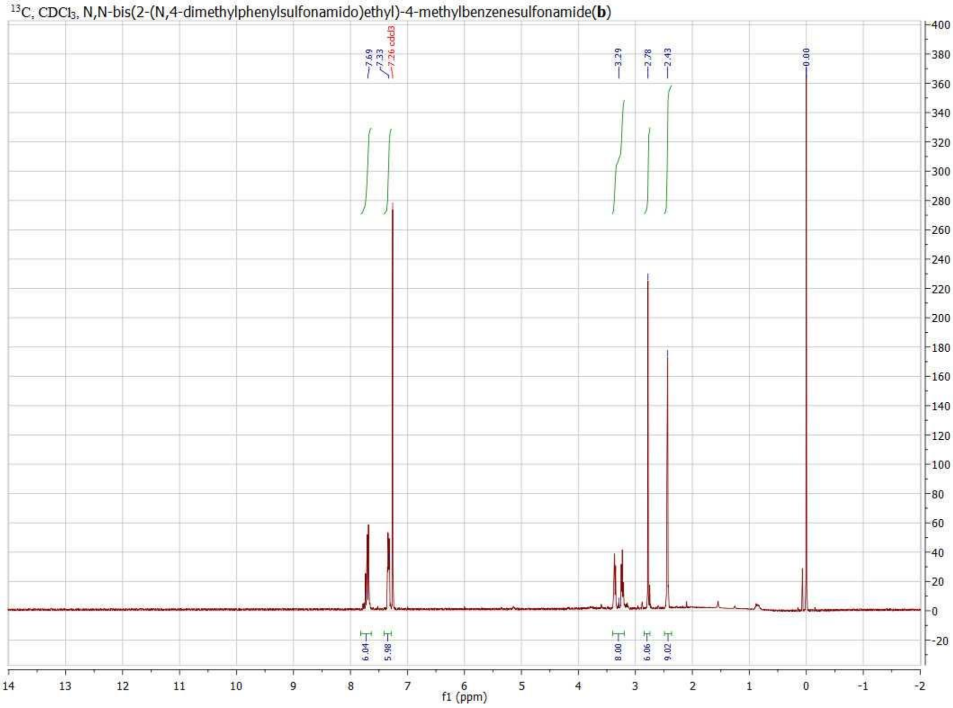

^{13}C NMR (400 MHz, $CDCl_3$):

N,*N*-Bis(2-(*N*,4-dimethylphenylsulfonamido)ethyl)-4-methylbenzenesulfon-amide (**2**)

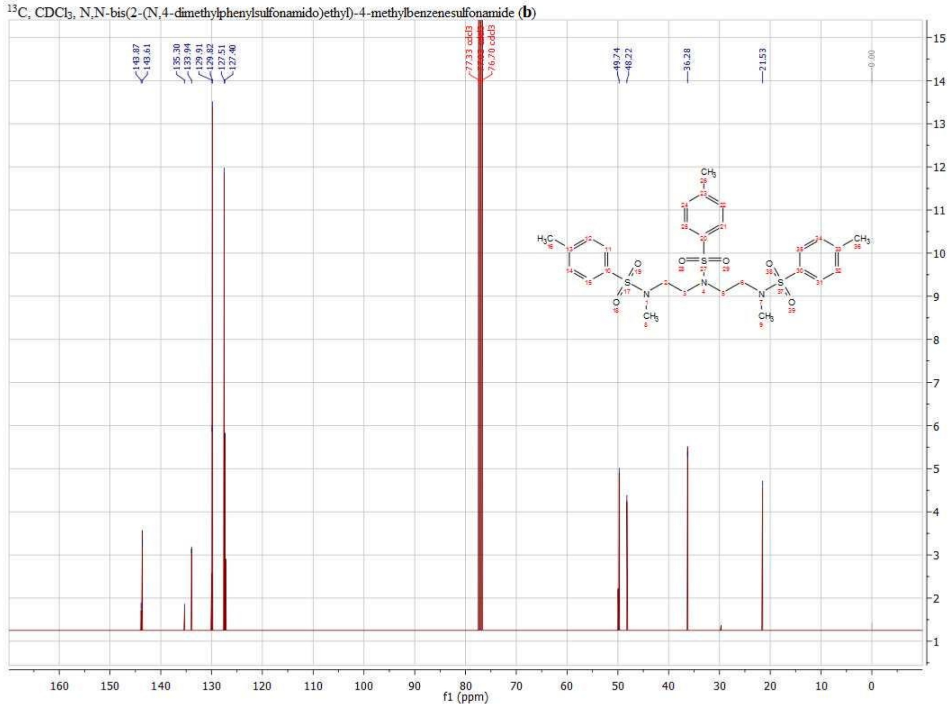

^{1}H NMR (400 MHz, D_2O):

N,*N*''-dimethyldiethylene triamine trihydrochloride (**3**)

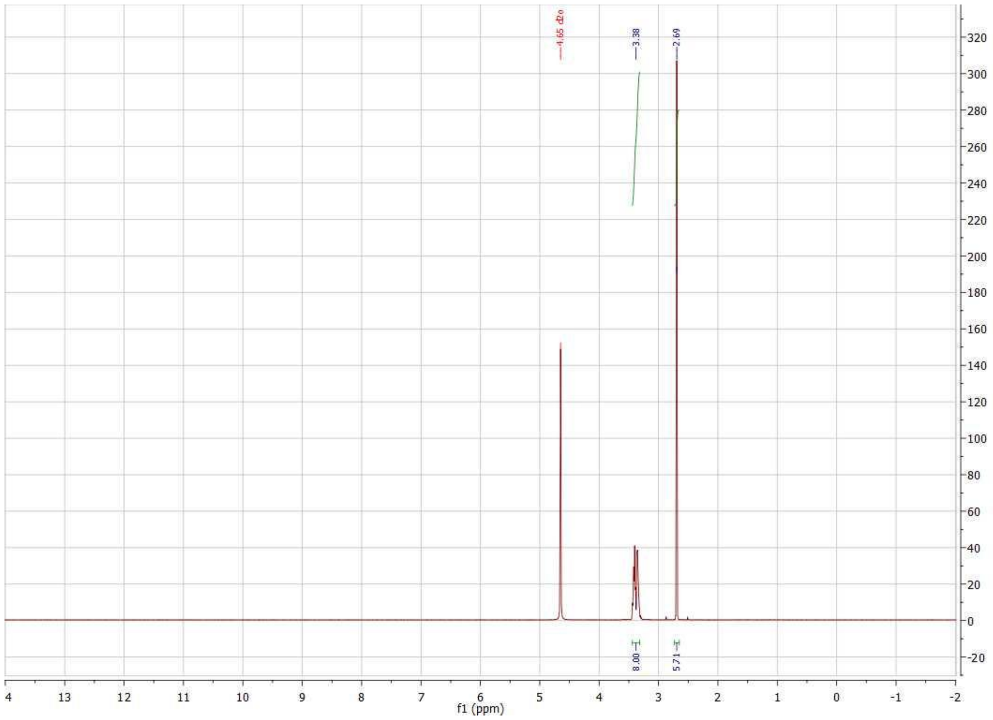

^{13}C NMR (400 MHz, D_2O):

N,*N*''-dimethyldiethylene triamine trihydrochloride (**3**)

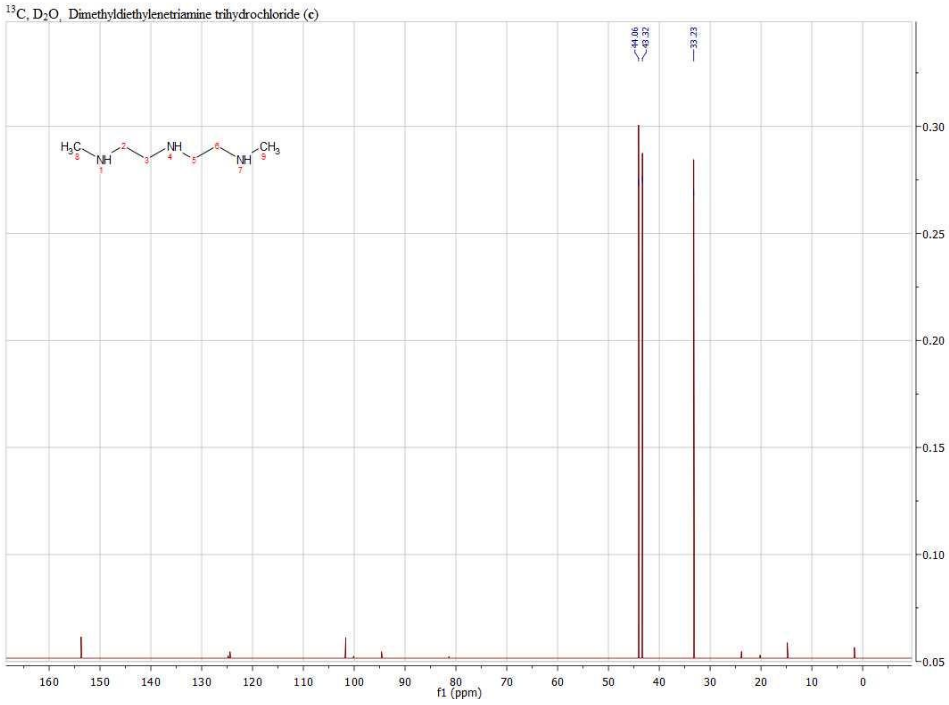

^{1}H NMR (400 MHz, $CDCl_3$):

N,*N*''-dimethyldiethylene triamine (**4**)

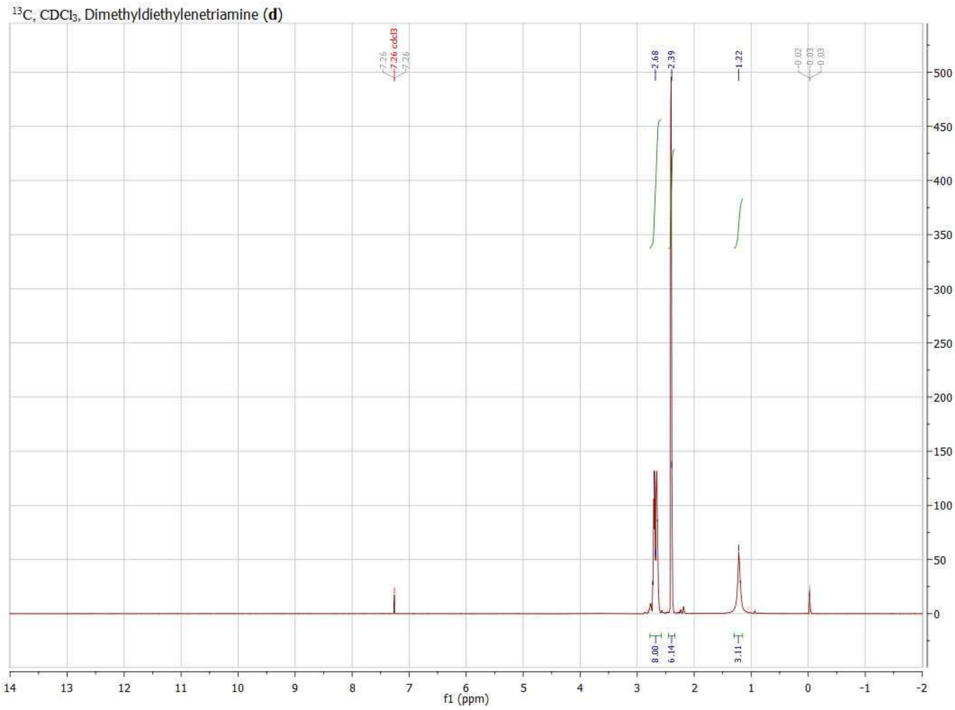

^{13}C NMR (400 MHz, $CDCl_3$):

N,*N*''-dimethyldiethylene triamine (**4**)

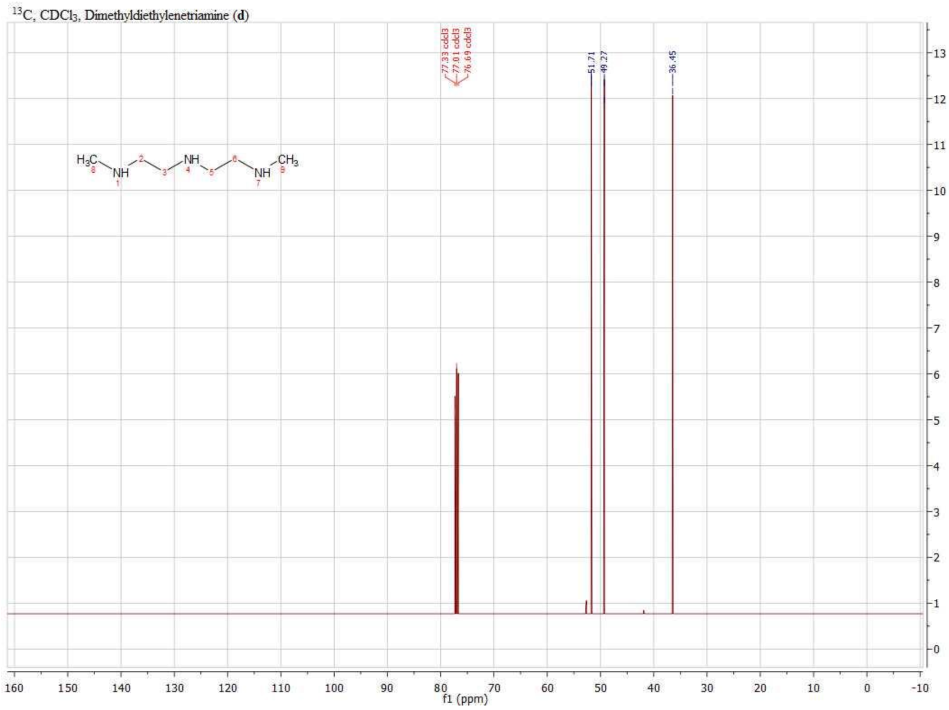

Supplement: File 1 — 1H NMR and 13C NMR data of compounds 1–4. [file Beilstein_J_Org_Chem-08-1909-s001.pdf]
